# Complete genome sequence of enterococcal phage G01

**DOI:** 10.1128/mra.01217-23

**Published:** 2024-01-31

**Authors:** Emma K. Sheriff, Shelby E. Andersen, Anushila Chatterjee, Breck A. Duerkop

**Affiliations:** 1 Department of Immunology and Microbiology, University of Colorado Anschutz Medical Campus, Aurora, Colorado, USA; DOE Joint Genome Institute, USA

**Keywords:** *Enterococcus*, bacteriophages, genomics

## Abstract

Here, we report the annotated genome of enterococcal phage G01. The G01 genome is 41,189 bp in length and contains 67 predicted open reading frames. Host range analysis revealed G01 can infect 28.6% (6/21) of *Enterococcus faecalis* strains tested and appears to not require the enterococcal phage infection protein PIP_EF_.

## ANNOUNCEMENT

Enterococci are minority members of the human microbiota; however, during antibiotic therapy, multidrug-resistant (MDR) enterococci can outgrow and cause opportunistic infection (1–6). MDR enterococcal infections are difficult to treat, leading to renewed interest in bacteriophages (phages) as a therapeutic option (7–12). Isolation and characterization of novel enterococcal phages will inform the rational development of targeted phage therapeutics.

Phage G01 was isolated from river water collected near the Adi Ganga Ghat, Kolkata, West Bengal, India (22.520498, 88.340097). The water was filtered (0.45 µm), and isolation was performed through serial passage on *Enterococcus faecalis* OG1RF in top agar, as previously described (13). G01 phages were amplified by infecting a culture of *E. faecalis* OG1RF and purified using cesium chloride gradient centrifugation (14). DNA was extracted using phenol:chloroform:isoamyl alcohol extraction (13). The same sample of G01 DNA was used for Illumina and Oxford Nanopore sequencing. Libraries were prepared with the Illumina DNA prep kit and IDT 10 bp UDI indices (Illumina) or the PCR-free Oxford Nanopore Technologies Ligation Sequencing Kit with NEBNext Companion Module (Oxford Nanopore). Illumina paired-end 150 bp sequencing was performed on a NextSeq 2000. Demultiplexing, quality control, and adapter trimming were performed with bcl-convert v3.9.3. Single-end Nanopore sequencing was performed on a MinION. Super-accurate basecalling, demultiplexing, and adapter removal were done using Guppy v6.4.6. Illumina and Nanopore sequencing reads were utilized for a hybrid assembly using SPAdes v3.15.5 (15, 16). Assembly resulted in one 38,867 bp contig and one 2,399 bp contig with coverages of 5572.3× and 6525.5×, respectively. The assembly also yielded 310 contigs of less than 283 bp with coverages less than 1.5×. Analysis proceeded with the two largest, highest coverage contigs. These contigs were mapped against a single 41,883 bp nanopore sequencing read using CLC Genomics Workbench v20.0.4 to determine orientation. Using PCR primers facing outward from each end of the assembled sequence, it was determined that the G01 genome is circularly permuted during replication (17, 18). Open reading frames were predicted using CPT Galaxy PAP Structural Workflow v2023.1 and annotated with RASTtk and InterProScan (19–23). All tools were run with default parameters.

**Fig 1:**
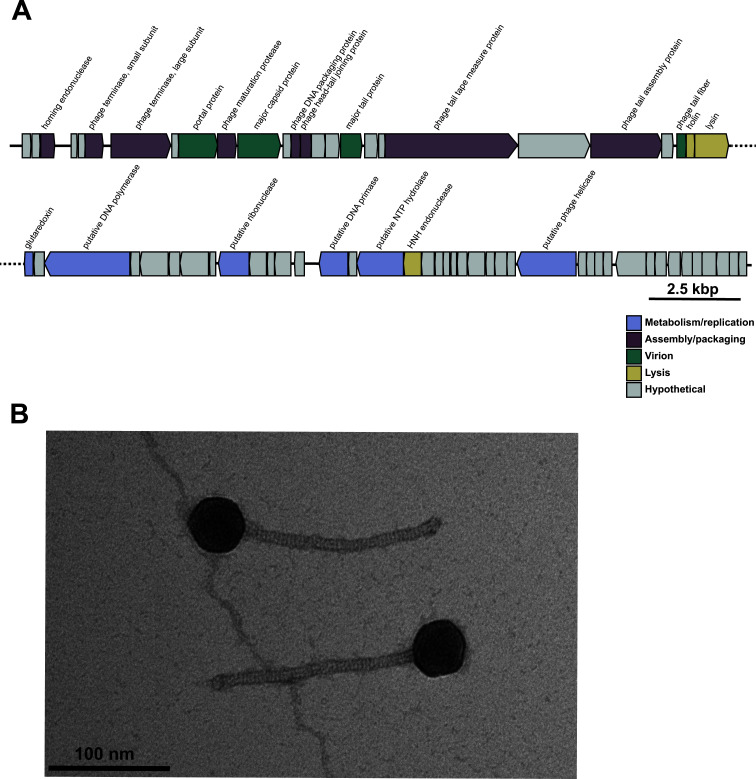
(**A**) The G01 genome contains 67 predicted open reading frames. Annotations are indicated above the open reading frame, if available, and colored by the predicted function. (**B**) Purified G01 virions were stained with 0.75% uranyl formate and subjected to transmission electron microscopy on a ThermoFisher Tecnai G2 12 BioTwin equipped with a side-mount XR80 (8mp) CCD AMT camera and a low-mount NS15B (15mp) sCMOS AMT camera, with an accelerating voltage of 80 kV. Micrographs of the G01 virion reveal it has siphophage morphology with a tail approximately 169 nm long and a capsid approximately 45 nm in diameter. Measurements reflect analysis of 17 individual virions using ImageJ (26).

The G01 genome is 41,189 bp in length with 34% GC content and 67 predicted open reading frames (Fig. 1A). G01 shares 95.56% nucleotide identity over 75% of its genome with the phage MSF2 (accession number MK982307), the most similar complete phage genome identified by BLAST and a member of genus *Efquatrovirus* (24). Transmission electron microscopy determined G01 is a non-contractile tailed phage with siphophage morphology (Fig. 1B). Host range analysis was performed on *E. faecalis* strains embedded in top agar as described previously (25). Of the 21 strains tested, G01 formed plaques on 28.6% of *E. faecalis* strains tested and caused growth inhibition at low dilutions for an additional six strains (Table 1). G01 apparently infects *E. faecalis* OG1RF independent of the phage infection protein PIP_EF_, a previously identified *E. faecalis* phage receptor that determines phage tropism (Table 1) (13). However, infection appears to depend on the enterococcal polysaccharide antigen that is required for phage adsorption (25).

**TABLE 1:** Dilutions of high titer G01 lysate were plated on *E. faecalis* strains for host range analysis^*a*^

*E. faecalis* bacterial strain	Infection phenotype	Reference
ARO1	None	(27)
ATCC 29212	None	(28)
ATCC 4200	2.7 × 10^10^ PFU/mL	(29)
CH188	None	(27)
Com6	1.1 × 10^11^ PFU/mL	(30)
D6	6 × 10^7^ PFU/mL	(27)
DS16	10^−1^	(31)
DS5	2.6 × 10^10^ PFU/mL	(27)
E1Sol	None	(27)
HIP11704	10^−1^	(27)
JH2-2	10^0^	(32)
Merz96	None	(27)
OG1RF	3 × 10^10^ PFU/mL	(33)
OG1RF∆*epaOX*	None	(34)
OG1RF∆*epaR*	None	(35)
OG1RF∆*pip*	5 × 10^10^ PFU/mL	(13)
SF28073	None	(36)
T1	10^−3^	(37)
T11	None	(27)
T2	6 × 10^10^ PFU/mL	(27)
T3	None	(27)
T8	10^0^	(27)
V583	None	(38)
X98	10^0^	(39)

^*a*^ If plaques formed on a given strain, the titer of the lysate on that strain is listed in the second column. If the strain showed growth inhibition at high dilutions without ever forming individual plaques, the lowest dilution in which clearing was seen is listed.

## Data Availability

Data are available at NCBI GenBank under the accession number OR797478. DNA sequencing reads have been deposited in the European Nucleotide Archive (ENA) under project accession number PRJEB68156. Illumina reads are deposited under run accession number ERR12205061. Nanopore reads are deposited under run accession number ERR12257447.
